# Case Report: Occurrence of Thyroid Storm in a Young Patient With Recurrent Diabetic Ketoacidosis

**DOI:** 10.3389/fendo.2022.834505

**Published:** 2022-03-15

**Authors:** Tatsuya Iino, Masayuki Akatsuka, Shuji Yamamoto

**Affiliations:** Department of Anesthesiology, Obihiro Kosei Hospital, Obihiro, Japan

**Keywords:** thyroid storm, diabetic ketoacidosis, young patient, occurrence, management

## Abstract

**Background:**

Thyroid storm (TS) is a fatal disease that leads to multiple organ failure and requires prompt diagnosis. Diabetic ketoacidosis (DKA) is a trigger for thyroid crisis. However, TS and DKA rarely occur simultaneously. Moreover, owing to the rarity of the co-occurrence, the clinical course remains unclear. In this study, we present a case of TS that developed during the follow-up for repeated DKA in a young patient.

**Case Presentation:**

A 25-year-old man with a history of recurrent DKA was brought to the emergency room frequently with similar symptoms. DKA treatment was initiated, but his tachycardia and disturbance of consciousness did not improve. Further examination of the patient revealed a Burch–Wartofsky Point Scale score of 80 points, consistent with the Japan Thyroid Association criteria. Therefore, DKA coexisting with TS was diagnosed. Antithyroid medication, inorganic iodine, and corticosteroids were then started as treatment for TS, and β-blockers were administered to manage tachycardia. With these treatments, the patient’s health improved and he recovered.

**Conclusions:**

In severe cases of recurrent DKA, the presence of TS should be considered, and early treatment should be initiated before the patient’s condition worsens.

## Introduction

Thyroid storm (TS) is a life-threatening endocrine emergency that leads to multiple organ dysfunction. The incidence of TS in Japan is 0.2 per 100,000 population per year (1). Undiagnosed and untreated TS in the late stages can be associated with high mortality (2, 3). Women are more likely to develop TS, which can occur at any age (4). TS usually results from precipitating events such as surgery, sepsis, burn injury, diabetic ketoacidosis (DKA), cardiovascular accidents, parturition, status epilepticus, I131 treatment, and iodinated contrast dyes (5).

DKA is a serious complication of a disordered metabolic state, characterised by hyperglycaemia, ketosis, and metabolic acidosis (6). The annual incidence of DKA is 0–56 per 1000 population per year (7). Women have a higher prevalence of DKA than men (7), and those aged 18–44 years are more likely to develop DKA than other age groups (8).

Previous reports have confirmed the co-occurrence of DKA and thyroid crisis. However, owing to its rarity, its clinical course remains unclear (9, 10). In this study, we present a rare case of TS in a young man with recurrent DKA.

## Case Description

### Ethics Consideration and Patient Details

This case study was conducted according to the CARE guidelines (11). Written informed consent was obtained from the patient for the publication of this case report and accompanying images.

A 25-year-old man who had a history of type 1 diabetes mellitus and was on insulin therapy was brought to the emergency room for disturbance in consciousness and nausea. The patient had been frequently admitted to the hospital for similar symptoms in the past few months and had a diagnosis of DKA because he had poor adherence to insulin therapy. He had a medical history of Graves’ disease since he was 15 years old. Methimazole and potassium iodide were used for the treatment. He frequently stopped going to the hospital at his own discretion. When the patient was admitted to the hospital for the aforementioned symptoms, 2 months had passed since his last discharge from the hospital.

### Clinical Finding

On admission, his vital signs were as follows: blood pressure, 130/64 mmHg; pulse rate, 220 beats per minute (atrial fibrillation); respiratory rate, 40 breaths per minute, O_2_ saturation, 99% on room air; body temperature, 36.3°C; E2, V3, M5, and a total of 10 points on the Glasgow Coma Scale. Results of blood gas analysis showed a pH of 7.036, glucose level of 736 mg/dL, base excess of -24.4 mmol/L, lactate level of 26.0 mg/dL, $HCO_{3}^{-}$ of 6.3 mmol/L, anion gap of 27.5 mmol/L, Na^+^ level of 125.6 mmol/L, K^+^ level of 5.15 mmol/L, and increased production of urine ketone body. The HbA1c level at the ICU admission was 12.2%. Considering the metabolic acidosis, hyperglycaemia, and the patient’s medical history, his condition was diagnosed as DKA, and he was therefore admitted to the intensive care unit.

### Treatment and Outcomes

Rapid administration of extracellular fluid to replenish intravascular volume, continuous administration of fast-acting insulin to manage hyperglycaemia, and correction of electrolyte abnormalities were performed, but he showed poor improvement in consciousness level.

Because of the prolonged disturbance of consciousness, a thorough examination was performed for a suspected diagnosis of thyrotoxicosis. Results of laboratory blood testing using electrochemiluminescent immunoassay revealed the presence of thyrotoxicosis, with the following clinical findings: thyroid-stimulating hormone, 0.005 μIU/mL [normal range: 0.50 – 5.0 μIU/mL]; free T3, 21 pg/mL [normal range: 2.3 – 4.0 pg/mL]; free T4, 6.7 ng/dL [normal range: 0.9 – 1.7 ng/dL].

In addition to the restlessness symptoms associated with the central nervous system, tachycardia, nausea, and congestive heart failure were observed, which are symptoms of TS. The patient’s medical history showed poor adherence with treatment for Graves’ disease, which also contributed to a suspicion of TS. Based on the Burch–Wartofsky Point Scale for TS (12), the patient’s score was 80, which was considered highly suggestive of TS. Based on the Japan Thyroid Association criteria (2), TS was diagnosed.

Antithyroid medication, inorganic iodine, and corticosteroids were promptly started as treatment for TS, and beta-blockers were administered for managing tachycardia. TS treatment was successful, and the patient’s consciousness disorder and tachycardia improved. DKA also improved without complications of other organ damage, and the patient was discharged to the general ward on the third day after admission to the intensive care unit. The patient was discharged on the tenth day of hospitalisation and continues to receive outpatient treatment. Progression of TSH, free T4, and free T3 during the clinical course is shown in **Table 1**.

**Table 1:** Progression of TSH, free T4, and free T3 during the clinical course.

	Day 1	Day 2	Day 3	Day 6	Day 16
**TSH (μIU/mL)**	0.005	0.005	0.005	0.005	0.005
**Free T3 (pg/mL)**	21.30	18.30	10.50	4.48	4.01
**Free T4 (ng/dL)**	6.70	7.22	6.65	4.33	2.68

Day 1: admission at the intensive care unit.

Day 2–Day 6: during the hospitalization.

Day16: outpatient.

TSH, thyroid-stimulating hormone; T3, triiodothyronine; T4, thyroxine.

## Discussion

This case reveals the clinical course of TS that developed during the follow-up of repeated DKA.

The occurrence of TS and DKA can be concurrent, which is rare but a life-threatening emergency presentation. A previous report showed that the mortality rate of the concurrent presentation of TS and DKA was 15% (13). While the mortality rate is high, the concurrent presentation of the two endocrine disorders, namely, DKA and TS, is very rare. Several factors could mask or delay early diagnosis. Therefore, the simultaneous presentation of these two endocrine emergencies poses a diagnostic challenge.

In this case, it was difficult to make a diagnosis of TS at the time of initial examination because the patient had a history of recurrent DKA, and the symptoms at the time of presentation were consistent with DKA. The symptoms associated with the central nervous system did not improve after initiating treatment for DKA, and because the patient had severe tachycardia and a history of hyperthyroidism, blood tests for assessing thyroid function were performed; these results led to the diagnosis of TS. TS could thus be diagnosed relatively early and early treatment could be started—one of the reasons the patient’s life could be saved.

Thyroid function and glucose metabolism are closely related, and normal thyroid function is essential for maintaining equilibrium in glucose metabolism. However, excess levels of thyroid hormones have been implicated to be responsible for increased intestinal glucose absorption, increased hepatic production of glucose from glycogen, decreased insulin secretion from the pancreas, increased insulin resistance, and increased renal clearance of insulin. A previous study showed that hyperthyroidism worsened glycemic control in patients with diabetes (14). Additionally, both TS and DKA have similar predispositions (15, 16), and they are likely caused by a common trigger. A previous report showed that an initial trigger by excessive levels of thyroid hormones causes DKA, which subsequently leads to TS (17). The coexistence of diabetes mellitus with hyperthyroidism is a known clinical observation and hyperthyroidism aggravates glucose intolerance *via* multiple mechanisms (18). A recent review shows that efforts should be made to maximize patient adherence to antithyroid and anti-diabetic agents when treating patients with the simultaneous development of TS and DKA (13). Another recent review demonstrates the importance of being aware of the possible simultaneous development of DKA and TS in patients with a history of Graves’ disease (19). In this case, the patient’s compliance with medication was poor, and inadequate antithyroid medication may have resulted in excess production of thyroid hormones, resulting in DKA and subsequent concurrent TS.

Based on our case findings, we suggest the following learning points. First, there needs to be awareness that DKA can precipitate TS. Second, if a patient with DKA presents with persistent tachycardia and disturbance of consciousness, TS should be considered in the differential diagnosis. Third, if the thyroid function tests revealed the diagnosis of thyroid crisis, the physician should start the management with antithyroid drugs, insulin and intravenous fluids.

## Conclusions

The simultaneous presentation of TS and DKA is rare, but it can be life-threatening if diagnosis and treatment are delayed. A comprehensive clinical evaluation, including physical examination and laboratory investigations, will be needed for the early detection of these rare presentations. We also need to pay close attention to the patient’s previous medication compliance to prevent deterioration of the concurrent presentation. These approaches could help clinicians in the early detection and multidisciplinary treatment of patients with concurrent TS and DKA.

## Data Availability Statement

The original contributions presented in the study are included in the article/supplementary material. Further inquiries can be directed to the corresponding author.

## Author Contributions

TI collected patient information and was responsible for preparing the first draft. MA was responsible for preparing the first draft and reviewing it. TI and MA analysed and interpreted patient information. SY supervised the project. All authors have read and approved the final manuscript.

## Funding

This research did not receive any specific grant from any funding agency in the public, commercial or not-for-profit sector.

## Conflict of Interest

The authors declare that the research was conducted in the absence of any commercial or financial relationships that could be construed as a potential conflict of interest.

## Publisher’s Note

All claims expressed in this article are solely those of the authors and do not necessarily represent those of their affiliated organizations, or those of the publisher, the editors and the reviewers. Any product that may be evaluated in this article, or claim that may be made by its manufacturer, is not guaranteed or endorsed by the publisher.

## Abbreviations

TS, Thyroid storm; DKA, Diabetic ketoacidosis.
